# Magnetic resonance imaging study determining cord level and occupancy at thoracolumbar junction in achondroplasia – A prospective study

**DOI:** 10.4103/0019-5413.73661

**Published:** 2011

**Authors:** Hitesh N Modi, Seung-Woo Suh, Jae-Young Hong, Jae-Hyuk Yang

**Affiliations:** Department of Orthopedics, Korea University Guro Hospital, South Korea

**Keywords:** Achondroplasia, cord and canal occupancy, cord compression, MRI, thoracolumbar kyphosis

## Abstract

**Background:**

Thoracolumbar (TL) stenosis in achondroplasia is frequently reported, and becomes symptomatic in adulthood. Hence we conducted a prospective study to determine cord level and occupancy at TL junction in symptomatic or asymptomatic achondroplasis patients in comparision to normal population by magnetic resonance imaging (MRI).

**Materials and Methods:**

Cord level with its occupancy rate and TL kyphosis were measured on MRI and standing radiogram, respectively. We prospectively studied MRI of TL spine in 19 patients (7 males and 12 females) with achondroplasia. All the subjects were randomly selected from our outpatient clinic and divided into two groups: symptomatic and asymptomatic group. Symptomatic group had at least two of the following symptoms: back pain with spasticity and walking difficulty, radicular pain in upper thigh or girdle pain, tingling and numbness in the lower limbs, visible deformity at TL spine and brisk reflexes in lower extremities. Asymptomatic group was selected from those patients who visited in outpatient clinic for consultation of limb lengthening. The third group was taken as control that comprised 11 nonachondroplasia otherwise normal patients (8 males and 3 females) who presented to our outpatient clinic for back pain.

**Results:**

Results showed spinal cord level was higher in achondroplasia than nonachondroplasia (*P*=0.003); however, no difference in cord level between symptomatic and asymptomatic group (*P*=0.568). Comparing cord occupancy, no difference found among all three groups (*P*=0.20). Kyphosis was increasing from nonachondroplasia, asymptomatic and symptomatic patient groups (*P*<0.001). Average age was 22.4±14.2, 11.9±6.5, and 36.2±13.2 years in symptomatic, asymptomatic, and nonachondroplasia groups, respectively (*P*<0.001).

**Conclusion:**

Our results indicated high level of spinal cord in achondroplasia patients compared to nonachondroplasia individuals. High prevalence of neurological symptoms at TL level in such patients can be associated with high cord level and developing progressive kyphosis at TL level along with degenerative process.

## INTRODUCTION

Neurological problems are present in 35–47% of patients with achondroplasia.^1^,^2^ There may be a delay in mental and motor development, hypotonia, feeding and sleep disorders, and compressive spinal syndromes in children; however, adults usually present with symptoms of spinal stenosis.^1^,^2^ These are associated with considerable disability and reduction in the quality of life. The cause of narrow spinal canal in achondroplasia is a disorder of enchondral ossification, which results in early fusion of the pedicles to the vertebral bodies at the neurocentral synchrondosis.^1^–^7^ Therefore, the cross-sectional area of spinal canal is consequently narrowed by shortened pedicles and decreased interpedicular distance, which leaves a reduced space available for the neural elements.^8^,^9^

Additionally, kyphosis at thoracolumbar (TL) junction is also a known phenomenon in patients with achondroplasia, which appears at the age of 6 months. TL kyphosis in majority of patients gets improved and only 10% will progress in kyphosis.^10^,^11^ Kyphotic deformity in achondroplasia patients has a higher risk of developing neurological complication due to relatively narrow spinal canal. The neurological signs of TL stenosis in achondroplasia patients are much more frequently reported and tend to manifest clinically in adulthood.^12^ There may be “wedge-shaped” deformities of one or more vertebral bodies, particularly between TI0 and L2 that exerts compression on the nervous structures and causes kyphosis of this segment of the spine particularly at conus and nerve roots.^13^ Fortuna *et al*. reported 23% of achondroplasia cases present with such complications.^12^ and reported three patients who were operated for laminectomy at TL junction having narrow canal.^8^ They believed that direct TL radiograms play an important role to diagnose this pathology. Kahanovitz *et al*.^14^ found various clinical-radiological correlations on the basis of plain radiograms alone; for example, TL kyphosis not related to age was found more frequently in more severely compromised patients while interpedicular distances of less than 2 cm at L1 and of less than 16 mm at L5 were found only in patients with severe paraparesis. However, not all achondroplesia patients having interpedicular distance less than 20 mm at L1 or conus level, would develop severe paraperesis. Recently, Modi *et al*.^15^ noted that even though the size of foramina narrowed in achondroplasia at lumbar level, the occupancy of lumbar nerve root is not more than normal patients, mainly due to narrowing of lumbar nerve root size. Therefore it is interesting to know that why achondroplasia patients do not develop severe paraparesis even though they show narrowing of spinal canal at TL junction along with kyphosis. We hypothesized that the cord occupancy in the spinal canal at TL junction remains the same compared to a normal individual. The reason for developing paraparesis in achondroplasia patients is mainly due to ligamentum flavum hypertrophy and/or instability at TL kyphosis, and not the spinal canal size.

## MATERIALS AND METHODS

The study was conducted to find out the cord level and occupancy at TL junction in symptomatic or asymptomatic achondroplastic in comparison to normal population by MRI and to find out if any differences exists. After approval from the institutional review board committee, we prospectively studied MRI of TL spine in 19 patients (7 males and 12 females) with achondroplasia. All the subjects were randomly selected from our outpatient clinic and divided into two groups: symptomatic and asymptomatic group. Symptomatic group had at least two of the following symptoms: back pain with spasticity and walking difficulty, radicular pain in upper thigh or girdle pain, tingling and numbness in the lower limbs, visible deformity at TL spine and brisk reflexes in lower extremities. We excluded those patients who had only radicular leg pain indicating lumbar canal. Asymptomatic group was selected from those patients who visited in outpatient clinic for consultation of limb lengthening. The third group was taken as control that comprised 11 nonachondroplasia otherwise normal patients (8 males and 3 females) who presented to our outpatient clinic for back pain. Written and informed consent were obtained from all the subjects before the study.

**Figure 1:**
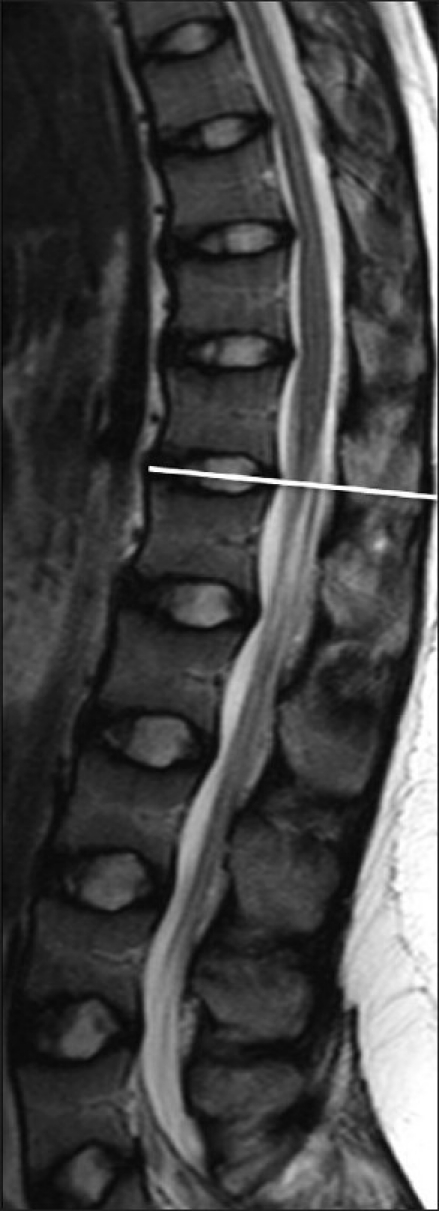
Sagittal T2 image of MRI in an achondroplasia patient comparing with axial image. It showed lowest level of spinal cord (conus medullaris) at T12-L1 disc

**Figure 2:**
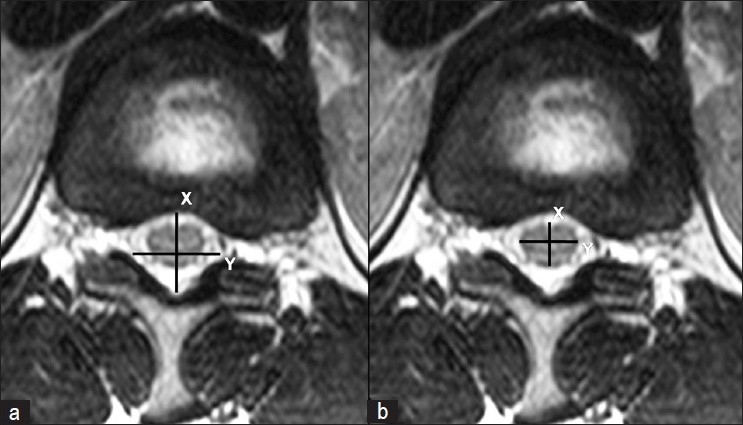
Measurement technique of anteroposterior (X) and horizontal (Y) diameter of (a) spinal canal and (b) conus medullaris at the widest portion of conus medullaris at thoracolumbar junction

All patients underwent anteroposterior and lateral radiogram of TL spine in standing position followed by MRI of the TL spine from T10-L5. MRI was performed using a 1.5 T scanner (Sonata; Siemens Medical System, Erlanger, Germany). Slices 3 mm thick were taken and measurements were made using a digital software program (Piview Star, Star Pacs Infinitt, Seoul, Korea). T1- and T2-weighted sagittal and axial cuts were obtained. All images were carried out by an experienced radiologist specializing in MRI of the spine. We found out the lowest level of the spinal cord (end of cord and beginning of cauda equina) to observe any discrepancy between achondroplasia and nonachondroplasia group. We marked the end of spinal cord by using reference point at pedicle or disc level [Figure 1]. We also measured anteroposterior (X) and horizontal diameter (Y) of the spinal canal and spinal cord, at the widest level of conus medularis diameter (at T11 or T12 level in all patients) in the axial image of MRI explained by Yukawa *et al*.^7^ [Figure 2]. We calculated the spinal canal area by a formula of measurement of an oval area using the equation X×Y×π/4 (height×width×π/4)^15^. Similarly, we also measured the area of conus medullaris (spinal cord) using the same formula. We measured the cord occupancy in percentage by dividing cord area with canal area. We have also measured kyphosis angle at TL junction on MRI as well as standing radiogram. All measurements were done by two fellows who were unaware about its purpose before the final draft. Both fellows measured all measurements two times with minimum interval of 2 weeks between two measurements. Interobserver and intraobserver reliability values r were 0.92 and 0.94, respectively.

We compared the difference in the level of spinal cord between symptomatic and asymptomatic achondroplasia group, between achondroplasia and nonachondroplasia patient group and among symptomatic, asymptomatic achondroplasia, and nonachondroplasia patient group using chi-square test. We have compared the height (X) and width (Y) of the spinal canal and spinal cord at T12 level between achondroplasia and nonachondroplasia group using student’s *t*-test. Similarly cord occupancy was also compared between both the groups using student’s *t*-test. We also analyzed the TL kyphotic angle among the symptomatic, asymptomatic, and nonachondroplasia groups to find out the relationship of back pain with kyphosis; and any difference in those groups with the control group using Mann-Whitney’s test. *P* value <0.05 was considered statistically significant for all tests.

## RESULTS

There were 9 and 10 achondroplasia patients in symptomatic and asymptomatic group, respectively. Average age was 22.4±14.2 (range, 9–50 years), 11.9±6.5 (range, 3–25 years), and 36.2±13.2 (range, 16–59 years) in symptomatic, asymptomatic, and nonachondroplasia patient groups, respectively, which is a statistically significant difference (*P*<0.001, ANOVA) in the age in all groups [Tables 1 and 2]. There were 9 achondroplasia patients who had symptoms of TL pain. Six out of them had TL kyphosis more than 30 degree, while 3 had less than 30 degree. Comparing the cord level in each group, it showed that in symptomatic group, 1, 6, and 2 patients had cord level at T12 pedicle, T12-L1 disc, and L1 pedicle level, respectively. Similarly in asymptomatic group 1, 4, 4, and 1 had T12 pedicle, T12-L1 disc, L1 pedicle, and L1-2 level, respectively, while in non achondroplasia group, 6 and 5 had L1 pedicle and L1-2 disc level. The level of spinal cord in achondroplasia appears to be higher than in nonachondroplasia patients. Comparing the level of cord using chi-square test, there is no significant difference between symptomatic and asymptomatic achondroplasia groups (*P*=0.568, chi-square); however, it was statistically significantly different when compared between achondroplasia and nonachondroplasia groups (*P*=0.0033, chi-square) or among symptomatic, asymptomatic achondroplasia, and nonachondroplasia groups (*P*=0.019, chi-square).

**Table 1:** Clinical details of symptomatic achondroplasia group, asymptomatic achondroplasia group and nonachondroplasia control groups

No	Age (years)	Sex (M/F)	Cord level	Canal height (mm)	Canal width (mm)	Canal area (mm^2^)	Cord height (mm)	Cord width (mm)	Cord area (mm^2^)	Cord occupancy (%)	TL kyphosis (°)
Symptomatic achondroplasia group											
1	9	M	L1 pedicle	12.2	18.8	180.05	6.9	9.3	50.37	27.98	31
2	35	M	L1 pedicle	12.3	18.8	181.52	7	11.8	64.84	35.72	26
3	18	M	T12 pedicle	9.2	15.2	109.77	5.3	6.7	27.88	25.39	47
4	10	F	T12-L1	11.5	16.5	148.95	6.2	9	43.80	29.41	36
5	17	F	T12-L1	12.3	18.8	181.52	5.6	7.1	31.21	17.19	28
6	11	M	T12-L1	14.7	13.3	153.48	9.7	10.7	81.48	53.09	52
7	35	F	T12-L1	10.1	15.1	119.72	6.6	7.1	36.79	30.73	59
8	17	F	T12-L1	15.2	18.2	217.16	8.4	10.1	66.60	30.67	30
9	50	M	T12-L1	10.9	17.3	148.03	8.6	9.2	62.11	41.96	21
Asymptomatic achondroplasia group											
10	14	F	L1 pedicle	11	18.7	161.47	6.7	9	47.34	29.31	34
11	3	M	L1 pedicle	11.7	15.2	139.60	6	7.2	33.91	24.29	18
12	25	M	L1 pedicle	12.1	16.1	152.93	6.9	11.5	62.29	40.73	12
13	10	F	L1 pedicle	11.1	18.2	158.59	7.2	8.7	49.17	31.01	30
14	13	F	L1-2	12.9	16.1	163.04	6.9	7.4	40.08	24.58	14
15	18	F	T12 pedicle	12.6	17.4	172.10	6.7	7.5	39.45	22.92	24
16	12	F	T12-L1	12.9	19.1	193.42	8.1	7.8	49.60	25.64	22
17	5	F	T12-L1	11.5	15.2	137.22	4.8	6.3	23.74	17.30	15
18	6	F	T12-L1	12.4	17.1	166.45	6.7	7.5	39.45	23.70	23
19	13	F	T12-L1	11.2	15.7	138.03	5.9	6.7	31.03	22.48	25
Nonachondroplasia control group											
20	39	M	L1 pedicle	10.6	19.3	160.60	10	6.2	48.67	30.31	5
21	59	F	L1-2	10.7	20.8	174.71	5.6	11.2	49.24	28.18	2
22	33	M	L1 pedicle	11.2	20.8	182.87	6.1	9.5	45.49	24.88	1
23	57	F	L1-2	13.1	19.3	198.47	6.8	7.5	40.04	20.17	3
24	16	M	L1-2	14.1	17.4	192.59	6.7	7.5	39.45	20.48	3
25	30	M	L1-2	15	20.2	237.86	7.5	9.2	54.17	22.77	1
26	44	F	L1 pedicle	13.8	16.7	180.91	6.9	8.6	46.58	25.75	5
27	30	M	L1 pedicle	12.2	15.1	144.61	5.8	7	31.87	22.04	12
28	31	M	L1-2	13.8	19	205.83	6.9	8.9	48.21	23.42	7
29	38	M	L1 pedicle	12.7	15.5	154.53	6.6	8	41.45	26.82	7
30	22	M	L1 pedicle	12.4	15	146.01	6.4	7.2	36.17	24.77	4

**Table 2:** Level of spinal cord in symptomatic and asymptomatic achondroplasia group and nonachondroplasia control groups along with their average age and numbers

Groups	n	Average age (years±SD)	End of spinal cord level				*P* value
			T12 pedicle	T12-L1 disc	L1 pedicle	L1-2 disc	
Symptomatic^a^	9	22.4±14.2	1	6	2	0	0.568^a/b^
Asymptomatic^b^	10	11.9±6.5	1	4	4	1	0.019^a/b/c^
Non-achondroplasia^c^	11	36.2±13.2	0	0	6	5	0.003^ab/c^

*P* value measure using chi-square test: a/b, symptomatic versus asymptomatic achondroplasia group; a/b/c, among all three groups; ab/c, achondroplasia versus nonachondroplasia patient groups

Comparing spinal canal area at conus medullaris level [Table 3], there is no statistically significant difference (*P*=0.15, ANOVA) found among symptomatic and asymptomatic achondroplasia and nonachondroplasia groups. Similarly comparing spinal cord area at the widest level of conus medularis, it did not show any significant difference (*P*=0.20, ANOVA) among all three groups. However, when cord occupancy is compared among all three groups, it exhibited statistically significant difference (*P*=0.045, ANOVA). Comparing the cord occupancy rate using Student’s *t*-test, it showed significant difference in the cord occupancy at TL level (*P*=0.025) between symptomatic and nonachondroplasia group, while there was no difference between symptomatic and asymptomatic groups (*P*=0.124) and asymptomatic and nonachondroplasia groups (*P*=0.442). When TL kyphosis were compared among all three groups, it showed significant difference in the TL kyphosis (*P*<0.001, ANOVA), with asymptomatic achndroplasic having 5 times more kyphosis and symptomatic achondroplasic patient having 8 times more kyphosis than normal control, which suggested that TL kyphosis in all three groups were different [Tables 1 and 3].

**Table 3:** Height (in mm), width (in mm), area (in mm^2^) of spinal canal and spinal cord at the widest level of conus medullaris, cord occupancy in percentage, and thoracolumbar kyphosis in all three groups

Groups	Spinal canal (mm/mm2)			Spinal cord (mm/mm^2^)			Cord occupancy (%)	Kyphosis (°)
	Height	Width	Area	Height	Width	Area		
Symptomatic	12.04±1.95	16.88±1.99	160.02±33.63	7.14±1.46	9±1.75	51.67±18.2	32.45±10.27	36.6±13
Asymptomatic	11.94±0.73	16.88±1.43	158.29±17.50	6.59±0.87	7.96±1.48	41.61±10.97	26.19±6.32	21.7±7.1
Non-achondroplasia	12.69±1.44	18.10±2.25	179.91±28.26	6.85±1.18	8.25±1.41	43.76±6.56	24.51±3.15	4.5±3.2

## DISCUSSION

The cause of a developmentally narrow lumbar canal in patients with achondroplasia is described as a growth disturbance and premature fusion of the posterior elements of the spine.^16^,^17^ The narrow spinal canal puts the spinal cord at risk for compression.^6^,^18^ In the present study, we observed that in achondroplasia patients spinal cord ends at a higher level than nonachondroplasia patients, which might be a one of the causative factor for frequent back pain and associated neurological symptoms in thoracolumbar and lumbar spine. Additionally, comparing the cord occupancy, spinal canal area, and cord area at conus medullaris level, it does not differ from nonachondroplsia subjects, which supports that the ratio of the size of the spinal canal and spinal cord remains the same as of non achondroplasic that should not be one of the responsible factor causing neurological symptoms.

The neurological signs of TL stenosis in achondroplasia patients are much more frequently reported and tend to manifest clinically in adulthood, although earlier than in degenerative stenosis in nonachondroplasia subjects without any sex difference.^12^ An earlier and more conspicuous onset of signs of degeneration with the growth of osteophytes that reduce the dimensions of the canal and/or the foramen even further; this occurs in 60% of cases of spinal stenosis.^12^,^14^ Another factor for stenosis is hypertrophy of the ligamentum flavum, which may or may not be calcified, and occurs fairly frequently (9% of cases).^14^ Lastly, there may be “wedge-shaped” deformities of one or more vertebral bodies, particularly between TI0 and L2. The retropulsed piece of vertebral body exert compression on the conus and the roots.^13^ In achondroplasia patients literature regarding TL spinal stenosis shows that symptoms mainly occur due to TL kyphosis that increase the risk to an already stenotic canal, and have a direct role in symptomatic spinal canal stenosis. If a developmentally narrow canal is the cause of the stenosis and its associated symptoms, the spinal cord or cauda equina should be compressed at the level of the vertebral body. In present study, we have included only those patients who had symptoms related to only TL stenosis having TL back pain with girdle pain, difficulty in walking with spasticity with neurological signs of upper motor neuron and not patients of lumbar stenosis causing radicular symptoms. Recently, Modi *et al*.,^15^ measured lumbar nerve root occupancy achondroplasia patients (n=17), and showed the similar occupancy in achondroplasia and the nonachondroplasia patients it seems the degenerative changes are the main cause for lumbar stenosis in such population. We have also measured various parameters of spinal canal and spinal cord at the widest level of the conus medullaris and thereby we calculated spinal canal and spinal cord area at that level. We have measured the canal width in AP plane from the posterior part of vertebral body to the anterior surface of laminae avoiding role of ligamentum flavum to prove that canal narrowing was not the responsible factor causing TL stenosis in such patients. We also calculated the percentage occupancy of spinal cord at TL junction where the spinal cord is the widest to observe any difference between achondroplasia and nonachondroplasia patient groups and found reduced occupancy as not the cause of neural deficit.

Level of spinal cord is an important factor regarding neurological problem. The spinal cord ischemia and paraplegia are major complications developing in achondroplasia patients. The reasons suggested were narrow spinal canal, ligamentum flavum hypertrophy, or degenerative osteophytes. Suzuki *et al*.^19^ recently reported a case report of 53-year-old achondroplasia man with flaccid paraplegia, which was developed due to ligamentum flavum hypertrophy and treated with laminectomy. He did MRI study; however he did not report regarding the level of cord. Therefore we have calculated the level of spinal cord ending in 19 (9 symptomatic and 10 asymptomatic) achondroplasia and 11 nonachondroplasia patients to compare any difference in the level of cord. The MRI study in achondroplasia patients focusing the lumbar spine have also been reported^9^,^15^. The spinal cord, which normally ends at the level of L1-2, were ending at T12-L1 or L1 pedicle level in most of cases with achondroplasia in the present study. We found that this higher level did not differ between symptomatic and asymptomatic achondroplasia patients (*P*=0.568) but it was significantly different from nonachondroplasia patients (*P*=0.003). So we believe that this higher level might cause some stretching effect on the cord or nerve root, which results into ischemic changes and neurological symptoms such as tingling and numbness. Modi *et al*.^15^ showed that in achondroplasia patients, the size of lumbar nerve root was relatively smaller than nonachondroplasia patients. We would think that it might be due to constant stretching effect over the nerve root or some other factors. Our finding of higher level of spinal cord did not differ in symptomatic and asymptomatic group, so the question remains why some patients have symptoms? Interestingly we have found that symptomatic group has higher age than asymptomatic group. Therefore we think that in asymptomatic group as age increases symptoms might develop later on. And therefore age and age-related degeneration process as well as ligamentum flavum hypertrophy play an important role along with higher level of cord in developing neurological symptoms.

Another important question is if really a narrow spinal canal size plays a major role in the development of symptomatology in achondroplasia patients. Modi *et al*.,^15^ in their study measuring the occupancy of lumbar nerve root, showed that the occupancy in these patients does not differ from nonachondroplasia and thus pointed out the degenerative process and development of osteophytes as a probable mechanism for neurological complication. In TL spine development of kyphosis and narrow spinal canal increases the risk of cord compression causing myelopathic features.^10^,^12^ We compared the cord occupancy using Kruskal Wallis test and it showed the lowest occupancy in nonachondroplasia group followed by asymptomatic and symptomatic achondroplasia group, respectively (*P*=0.045). The symptomatic achondroplasia group had significantly higher occupancy than nonachondroplasia group (*P*=0.025). In asymptomatic group cord occupancy rate did not differ from nonachondroplasia group (*P*=0.442). Our findings proved that in achondroplasia patients, symptoms do not appear due to narrow spinal canal as it was not different from nonachondroplasia group; however, as age increases (in symptomatic group) symptoms appear due to ligamentum flavum hypertrophy and other degenerative processes and the increased cord occupancy produces symptoms at TL level. Another factor increasing cord occupancy rate in achondroplasia group is TL kyphosis as it was significantly different even in symptomatic and asymptomatic achondroplasia patients groups (*P*=0.005).

The limitation of this study is small sample size with wide range of age in each group; we still feel that our findings would definitely throw more focus on this very rare disease, which has higher incidence rate of spinal stenosis as well as TL spinal deformity. However, same study with a larger numbers of patients, if possible, would further validate our findings. The randomizing achondroplasia patients with only TL stenosis symptoms are not easy which restricted our subject allocation to less numbers in spite of our institution is attached with achondroplasia society. Due to limitation of financial resources we could not measure the canal diametre by CT scan and to find out the cord level simultaneously, we investigated with MRI.

## CONCLUSION

The achondroplasia patients have relatively higher level of cord level than nonachondroplasia patients, which may be one of the important factor producing neurological symptoms possibly due to stretching effect and symptoms of spinal stenosis do not appear due to narrow bony/spinal canal, rather it remains the same as nonachondroplasia patients. Symptoms of spinal stenosis appears to happen due to degenerative process and increasing TL kyphosis.

This study would further point out that even though the achondroplasia patients do not have symptoms at presentation, symptoms can appear as age increases and/or kyphotic deformity increases, which are the only factors that matter in producing spinal stenosis.
